# Anti-PD-1 Monotherapy in Advanced Melanoma—Real-World Data from a 77-Month-Long Retrospective Observational Study

**DOI:** 10.3390/biomedicines10071737

**Published:** 2022-07-19

**Authors:** Daniella Kuzmanovszki, Norbert Kiss, Béla Tóth, Tünde Kerner, Veronika Tóth, József Szakonyi, Kende Lőrincz, Judit Hársing, Eleonóra Imrédi, Alexa Pfund, Ákos Szabó, Valentin Brodszky, Fanni Rencz, Péter Holló

**Affiliations:** 1 Department of Dermatology, Venereology and Dermatooncology, Faculty of Medicine, Semmelweis University, 41 Mária u., H-1085 Budapest, Hungary; kiss.norbert@med.semmelweis-univ.hu (N.K.); toth.bela@med.semmelweis-univ.hu (B.T.); kerner.tunde_zsuzsanna@med.semmelweis-univ.hu (T.K.); toth.veronika@med.semmelweis-univ.hu (V.T.); szakonyi.jozsef@med.semmelweis-univ.hu (J.S.); lorincz.kende@med.semmelweis-univ.hu (K.L.); harsing.judit@med.semmelweis-univ.hu (J.H.); nardaine_imredi.eleonora@med.semmelweis-univ.hu (E.I.); pfundalexa@gmail.com (A.P.); hollo.peter@med.semmelweis-univ.hu (P.H.); 2 Department of Health Policy, Corvinus University of Budapest, 8 Fővám tér, H-1093 Budapest, Hungary; akos.szabo@uni-corvinus.hu (Á.S.); valentin.brodszky@uni-corvinus.hu (V.B.); fanni.rencz@uni-corvinus.hu (F.R.); 3 Károly Rácz Doctoral School of Clinical Medicine, Semmelweis University, 26 Üllői út, H-1085 Budapest, Hungary

**Keywords:** anti-PD-1 monotherapy, nivolumab, pembrolizumab, advanced melanoma, immune checkpoint inhibitor, adverse event

## Abstract

Real-world evidence plays an important role in the assessment of efficacy and safety of novel therapies. The increasing use of immune checkpoint inhibitors (ICIs) in patients with advanced melanoma has led to notably improved clinical outcomes, while they are also associated with immune-related adverse events (irAEs). The majority of the available data are based on clinical trials, where the investigated subjects often do not adequately represent the general patient population of the everyday practice. Although there is a niche of objective biomarkers for the future treatment response of ICIs, certain studies suggest that irAEs may be predictive. The aim of this study was to carry out a retrospective analysis of treatment data from patients with advanced melanoma, treated with a single anti-PD-1 agent (pembrolizumab or nivolumab) during a 77-month-long period. Treatment efficacy and occurrence of adverse events were analyzed to identify potential predictive markers. Primary and secondary endpoints were the overall survival (OS) and progression-free survival (PFS). In our cohort, we demonstrated that the occurrence of more than one irAE showed a correlation with response to PD-1 ICI therapy and improved the OS and PFS. Our study suggests, that the grade of toxicity of the irAE may affect the survival rate.

## 1. Introduction

Over the last decade, the treatment of advanced melanoma (AM) has undergone a substantial change. The introduction of immune checkpoint inhibition (ICI) and targeted therapies has substantially improved the clinical outcomes of AM patients [1,2,3,4]. In this new era, ipilimumab, a monoclonal antibody targeting the cytotoxic T-lymphocyte antigen (CTLA)-4, was the first ICI therapy that provided an improvement in overall survival (OS) [4,5]. The use of ipilimumab resulted in a long-term survival in one fifth of the patients, with the majority of them achieving ten-year survival [5]. However, its use is limited by severe immune-related adverse events (irAEs) [6,7,8].

Following the approval of ipilimumab, novel ICIs targeting programmed cell death (PD)-1 were also approved for the treatment of AM. The programmed death ligand 1 (PD-L1) protein is expressed in the tumor cell membrane [9,10,11]. Upon the binding of its ligand, PD-1, which is expressed in activated T cells, NK cells, regulatory T cells and B cells, it inhibits the proliferation and cytotoxic effects of T cells in order to promote immune escape. This leads to the restriction of T-cell-mediated immune responses that may result in recurrent disease and metastases. Nivolumab, a human monoclonal IgG4 kappa antibody, and pembrolizumab, a humanized monoclonal immunoglobulin IgG4 antibody, block PD-1 and restore the immune response of T cells and enhance the recognition of tumor cells by the activated T cells. Nivolumab and pembrolizumab were approved by the FDA in 2014 for patients with AM who did not respond to first-line treatment [6,12]. In 2015, they were approved as first-line therapy, reaching 26–40% response rates in AM patients, providing higher survival rates than chemotherapy [1,6,8]. Further evidence showed that PD-1 ICI provide higher efficacy and a more tolerable safety profile [5,6,12]. They provide a long-term response, while AEs are usually manageable with the use of systemic corticosteroids or other immunomodulators, treatment cessation and supportive care [5,6,12,13]. 

In addition, PD-1 ICIs were recently also approved for stage III melanoma as a novel adjuvant treatment option. Their administration significantly decreases the hazard ratios of melanoma recurrence compared to placebo treatment and also ipilimumab [4]. 

Ipilimumab and nivolumab can be also given in combination therapy, which provides a higher objective response rate. However, the safety profile of this combination is less favorable and it does not result in a significant survival benefit compared to PD-1 ICI monotherapy [3,8]. 

BRAF and MEK inhibitors are small molecules that are used in combination treatment, which can offer a survival benefit for around the half of those AM patients who have an activating BRAF mutation [2]. While they usually provide a quick response, within a one year period, the majority of patients unfortunately develop therapy-resistant disease [2,4].

Novel immunotherapies for AM are approved based on the clinical outcomes of phase III studies, which require carefully determined inclusion and exclusion criteria, e.g., patients with an ECOG (Eastern Cooperative Oncology Group) status of ≥2, and those with active brain metastases or autoimmune diseases were excluded. However, the majority of patients in the everyday practice with AM do not fit these strict eligibility criteria. Moreover, significant differences in the median OS were reported in patients who met these eligibility criteria, in contrast to those patients who did not (18.3 vs. 5.43 months) [7]. Thus, the observed outcomes from phase III trials may not correlate with real-world clinical evidence in terms of efficacy and safety. Overall, the survival of AM patients has shown a significant increase since ICI and BRAF plus are used, based on the results of registry studies [5,14].

Here, we evaluated the efficacy and safety of an out-of-trial, real-life population of AM patients treated with anti-PD-1 monotherapy with nivolumab or pembrolizumab. 

## 2. Materials and Methods

A retrospective descriptive analysis of patients with unresectable stage III (M0) or stage IV melanoma (M1a–d) who were undergoing treatment with a single PD-1 inhibitor agent, pembrolizumab or nivolumab, at our department was carried out. We analyzed a 77-month period from 1 May 2015 to 31 October 2021. We collected patient data from our electronic health records (e-MedSolution, T-Systems Hungary, Budapest, Hungary).

Inclusion criteria were the following: the presence of metastatic or unresectable cutaneous AM that was treated with PD-1 ICI monotherapy during the study period, for at least two cycles of the standard dosing of nivolumab (3 mg/kg every 2 weeks) or pembrolizumab (2 mg/kg every 3 weeks). Exclusion criteria were patients with primary uveal melanoma and primary mucosal melanoma, and patients with incomplete medical records. The data cutoff was set to 30 June 2021, giving a minimum follow-up of four months. 

The patients received either nivolumab or pembrolizumab administrated intravenously at our outpatient clinic. The dosage of nivolumab was 3 mg/kg every second week, or a 240 mg flat dose every two weeks, or a 240 mg flat dose every four weeks. The dosage of pembrolizumab was 2 mg/kg every three weeks, or a 200 mg flat dose every three weeks, or a 400 mg flat dose every six weeks. 

The choice of PD-1 ICI and its dosage was selected based on the availability of the drug and the Hungarian AM treatment guideline, that changed during the investigated period given its long duration. PD-1 ICI treatment was administered until disease progression, unacceptable toxicity, death of the patient, or if the treating physician decided to withdraw the drug. Patients who had received prior treatment with BRAF/MEK inhibitors, prior treatment with whole brain radiotherapy (WBRT), or superficial radiation therapy were also included. 

The patients’ demographic data, subtype of the primary melanoma, disease stage at first presentation, BRAF gene mutation status and lactate dehydrogenase (LDH) level data were collected from the electronic health records, as well as baseline characteristics before PD-1 ICI therapy initiation. 

The treatments used, their duration, efficacy based on imaging (MRI, CT) and clinical evaluation, and the reasons for treatment cessation were analyzed. 

Data collection for the analysis of treatment-related AEs including the grade of severity and type of AE was carried out based on the common terminology criteria for adverse events (CTCAE) classification v.5.0. 

### Statistical Analysis

We determined real-world OS (rwOS) based on the start date of anti-PD-1 therapy and the date of the demise or last follow up of the patient. Real-world progression-free survival (rwPFS) was calculated from the start of the treatment until disease progression or death, or, if there was no progression, until the last follow-up date. The Mann–Whitney U test or Fisher’s exact test was used for descriptive analysis, as applicable. RwOS and rwPFS rates were estimated using the Kaplan–Meier method and differences between survival curves for each risk factor were compared by log-rank test. Parameters significant in univariate evaluation were selected for multivariate Cox proportional hazard regression analysis. The CI was set to 95% and a *p* value <0.05 was considered statistically significant. All analyses have been conducted with the IBM SPSS Statistics for Windows software, Version 25.0. (IBM Corp, Armonk, New York, NJ, USA).

## 3. Results

### 3.1. Patient Characteristics

At the start of data collection, 126 patients treated at our department with a single agent PD-1 ICI were identified. Three patients had ocular melanoma and four patients had mucosal melanoma and were excluded from the analysis. Consequently, 119 patients were analyzed, who were treated with the PD-1 ICI nivolumab or pembrolizumab between 1 May 2015 and 31 October 2021. Demographic and clinical characteristics at baseline are detailed in Table 1. The median duration of follow-up from treatment initiation was 45.1 weeks (range 17.3–112.1). The median number of the doses of pembrolizumab or nivolumab was 14 (range 7–32). The median age was 69 years, and 68 (57.1%) patients were male. The majority of the patients were ECOG PS 0-1 (117, 98.28%), only two patients had ECOG performance status ≥ 2. Nine (7.56%) patients had an elevated LDH level at the beginning of the anti-PD-1 therapy. A total of 52 (43.7%) patients received nivolumab treatment, while 66 (55.5%) were treated with pembrolizumab. In one case, the initiation of nivolumab resulted in a severe adverse skin reaction (grade 3). Therefore, nivolumab was discontinued and the patient continued the treatment with pembrolizumab. A total of 81 (68.1%) patients were treatment-naïve (received first-line treatment), while 38 (31.9%) received anti-PD-1 therapy as a second- or third-line treatment. In 56 cases (47.04%), we identified M1c status at the initiation of the treatment. 

The histopathological characteristics of the primary melanoma were as follows: 36 patients (30.3%) had pT4b melanoma and 35 patients (29.4%) had pTx (in which the Breslow score of the primary tumor cannot be assessed) or occult melanoma. Based on tumor-infiltrating lymphocytes (TIL) within the primary melanoma, we found 15 (12.6%) were brisk and 74 (62.2%) remained unknown. The nodular melanoma subtype was identified in 33 cases (27.7%) and the superficial spreading melanoma subtype and those not classified made up 23 cases (19.3%). A total of 98 patients (82.32%) had primary cutaneous melanoma and 21 patients (17.65) had occult primary melanoma. Twenty-five patients (21%) had brain metastases at the time of treatment initiation (Table 2).

Eighty-one patients (88, 73.9%) had BRAF wild-type melanoma, whereas only 31 (26.1%) patients harbored the BRAF V600 mutation. Only seven (5.88%) patients with BRAF-positive melanoma received nivolumab or pembrolizumab PD-1 ICI as first-line treatment (Table 3). A total of 24 (20.16%) patients who harbored the BRAF W600 mutation were previously treated with a BRAF-MEK inhibitor. Immune-related adverse events occurred in 55 (46.3%) patients, of whom, 31 (26.1%) patients had more than one irAE (Table 6). The reasons for treatment discontinuation were disease progression in 49 patients (41.2%), unacceptable toxicity in 13 patients (10.9) and complete response (CR) in two patients (1.7%) (Table 1). Nineteen (16%) patients died during the investigated period, of whom, two demised as the result of COVID-19 complications.

### 3.2. Efficacy–Survival Data 

The objective response rate (ORR), defined as the rate of patients with a complete response (CR) and partial response (PR), was only 16.8%. The disease control rate (DCR), defined as rate of patients with CR, PR and stable disease (SD), was 52.08% (Table 3). The treatment-naïve patients and the patients not treated as first-line had an almost similar ORR (17.2%, 15.78%) as the whole investigated population’s ORR. 

The ORR for BRAF wild-type patients was 19.31%, and the ORR for BRAF mutant patients was only 9.67. However, from 31 patients with a BRAF V600 mutation, only seven received first-line anti-PD-1 therapy. Among the patients harboring a BRAF mutation, CR occurred in two cases and PR only in one case.

The differences in demographics and clinical characteristics of the responders (*n* = 20), those AM patients who achieved an objective response; and non-responders (*n* = 89), patients who achieved SD or PD as the best response, were compared. Outcome data for one patient was not available (NA) (Table 1). The association of the examined parameters with survival outcomes was also investigated (Table 4).

**Figure 1:**
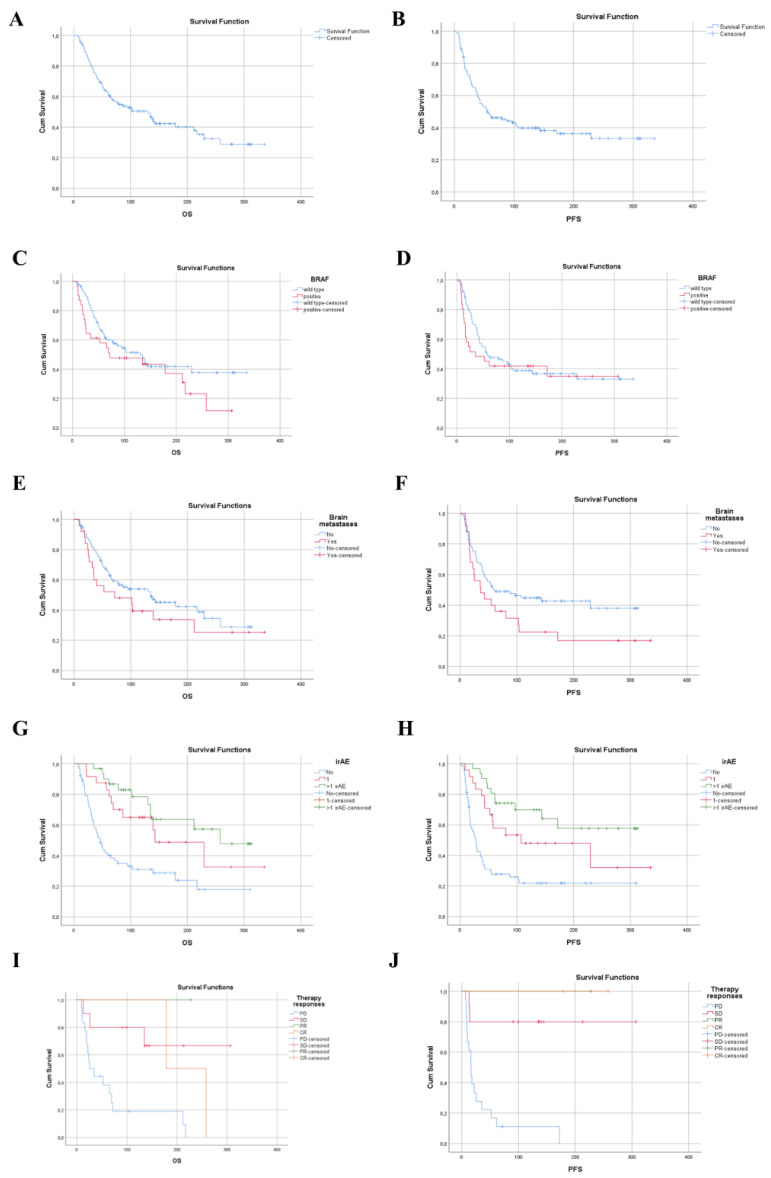
Kaplan–Meier survival estimates the survival. (**A**) OS in whole population, (**B**) PFS for whole population, (**C**) OS according to BRAF status, HR for death for patients with BRAF mutation was 1.96 (95% CI 1.02–3.77, *p* = 0.004), (**D**) PFS according to BRAF status, HR for progression for patients with BRAF mutation was 2.68 (95% CI 1.26–5.72, *p* = 0.011), (**E**) OS according to presence of brain metastases, HR for death 0.84 (95% CI 0.09–7.73, *p* = 0.880), (**F**) PFS according to presence of brain metastases, HR for progression 1.62 (95% CI 0.18–14.25, *p* = 0.666), (**G**) OS according to irAE, HR for death 0.3 (95% CI 0.14–0.64, *p* = 0.02), (**H**) PFS according to irAE, HR for progression 0.26 (95% CI 0.13–0.54, *p* = 0.000), (**I**) OS according to response of therapy, HR for death 0.14 (95% CI 0.05–0.43, *p* = 0.001), (**J**) PFS according to response of therapy, HR for progression 2.06 (95% CI 0.24–17.84, *p* = 0.511). OS—overall survival, PFS—progression-free survival, HR—hazard ratio, NR—not reached, CR—complete response, PR—partial response, SD—stable disease, PD—progressive disease.

The whole population median OS was 130 weeks (30 months) (Figure 1a) and the PSF was 54 weeks (12.5 months) (Figure 1b). 

In patients under 70 years of age, the median OS was 135 weeks; for patients ≥70 years old, the median OS was 86 weeks. The mPFS was almost same in these two groups (54.5 weeks, *p* = 0.982) (Table 4).

The patients with a BRAF-mutant melanoma had a shorter median OS and PFS compared to patients with BRAF wild-type disease; median OS was 71 weeks vs. 130 weeks (HR for death for patients with a BRAF mutation was 1.96, 95% CI 1.02–3.77 *p* = 0.004, Table 5) and median PS was 35 weeks vs. 58 weeks (HR for progression 2.68, 95% CI 1.26–5.72 *p* = 0.011) (Figure 1c,d). 

BRAF-mutant melanoma patients, who were previously treated with a BRAF inhibitor (with or without the addition of a MEK inhibitor) and received the PD-1 ICI not as first line therapy, had a longer survival compared to patients with a BRAF mutation and no previous BRAF inhibitor treatment (mPFS was 61 weeks vs. 12 weeks, *p* = 0.022) (Table 4). 

That patients who had no brain metastases had a longer OS and PFS compared to those who had brain metastases (median OS 135 weeks vs. 71 weeks; HR for death for patients with brain metastases was 0.84, 95% CI 0.09–7.73, *p* = 0.880, median PFS 61 weeks vs. 36 weeks; HR for progression 1.62, 95 CI 0.18–14.25, *p* = 0.666 Figure 1e,f, Table 4 and Table 5).

We noticed that patients who had more than one irAE (>1irAE) had a longer OS and PFS compared to those who did not have an irAE (median OS was 258 weeks vs. 46 weeks, respectively (HR for death 0.21% CI 0.10–0.44 *p* < 0.001, median PFS was not reached; HR for progression 0.17 95% CI 0.08–0.35 *p* < 0.001, Figure 1g,h, Table 4 and Table 5).

The presence of a grade 3–4 irAE meant a better outcome (HR for death 0.14, 95% CI 0.02–1.03, *p* = 0.053, HR for progression 0.11, 95% CI 0.01–0.89, *p* = 0.038).

The patients who was simultaneously treated with radiotherapy and ICI monotherapy showed a prolonged OS (*p* < 0.010) (HR for death 0.48 (95% CI 0.24–0.98, *p* = 0.43, Table 4).

### 3.3. Toxicity

An immune-related AE (irAE) occurred in a total of 55 patients (46.2%). A mild irAE (grade 1–2) developed in 37 patients (31.08%), while a severe irAE (grade 3–4) developed in 18 patients (15.12%) (Table 6). There were no treatment-related deaths. The most frequently affected organ system was the hepato-pancreato-biliary system (26.15%), endocrine (23.52%) system and gastrointestinal system (19.32%). The most frequent grade 3–4 irAEs were colitis (9.24%) and pneumonitis (6.72%). Due to an irAE, 42 patients (35.28%) required immune-modulation treatment (mainly systemic steroids and, in two cases, infliximab). AEs led to permanent treatment discontinuation in 13 patients (10.92%).

**Table 6:** The occurrence of immune-related adverse events among the investigated patients.

IrAE	Grade 1–2 N = 37 (31.08%)	Grade 3–4 N = 18 (15.12%)	All Grade N = 55 (46.2%)
Endocrine	24 (20.16)	4 (3.36)	28 (23.52)
hypothyroidism	16 (13.44)	-	16 (13.44)
hyperthyroidism	2 (1.68)	-	2 (1.68)
hypopituitarism	6 (5.04)	4 (3.36)	10 (8.4)
respiratory	1 (0.84)	8 (6.72)	9 (7.56)
pneumonitis	1 (0.84)	8 (6.72)	9 (7.56)
Gastrointestinal	9 (7.56)	14 (11.76)	23 (19.32)
colitis	5 (4.2)	11 (9.24)	16 (13.44)
gastritis	4 (3.36)	2 (1.68)	6 (5.04)
terminalis ileitis	-	1 (0.84)	1 (0.84)
Hepato-pancreato-biliary	25 (21)	6 (5.04)	31 (26.04)
hepatitis/ALT elevated	10 (8.4)	2 (1.68)	12 (10.08)
bilirubin elevated	6 (5.04)	-	6 (5.04)
pancreatitis	8 (6.72)	3 (2.52)	11 (7.56)
hyperlipidemia	1 (0.84)	1 (0.84)	2 (1.68)
Musculoskeletal	9 (7.56)	2 (1.68)	11 (7.56)
myositis	5 (4.2)	2 (1.68)	7 (5.88)
arthritis	4 (3.36)	-	4 (3.36)
Renal	5 (4.2)	4 (3.36)	9 (7.56)
nephritis	5 (4.2)	4 (3.36)	9 (7.56)
Skin	12 (10.04)	3 (2.52)	15 (12.6)
vitiligo	5 (4.2)	-	5 (4.2)
dermatitis	5 (4.2)	3 (2.52)	8 (6.72)
bullous pemphigoid	2 (1.68)	-	2 (1.68)
Nervous system	4 (3.36)	1 (0.84)	5 (4.2)
polyneuropathy	4 (3.36)	-	4 (3.36)
encephalitis	-	1 (0.84)	1 (0.84)
Hematological	1 (0.84)		1 (0.84)
pancytopenia	1 (0.84)	-	1 (0.84)
Ophthalmic	3 (2.52)	1 (0.84)	4 (3.36)
bulbitis	-	1 (0.84)	1 (0.84)
conjunctivitis	1 (0.84)	-	1 (0.84)
uveitis, iridocyclitis	2 (1.68)	-	2 (1.68)
Oral cavity/ear	4 (3.36)		4 (3.36)
periodontitis	1 (0.84)	-	1 (0.84)
otitis media, otitis externa, sinusitis, ethmoiditis	3 (2.52)	-	3 (2.52)

## 4. Discussion

The present study has been conducted at a single center between 2015 and 2021, and includes the retrospective data analysis of patients with AM treated with anti-PD-1 monotherapy with nivolumab or pembrolizumab. 

This cohort consisted of 119 patients with known unfavorable characteristics such as brain metastases (stage M1d disease), being older individuals (≥70 years old), poor performance status (≥2 ECOG), prior treatment, BRAF V600 mutation and the presence of an active autoimmune disorder requiring systemic steroid treatment. The majority of these patients would not meet the inclusion and exclusion criteria of the respective phase III trials [6,15].

In our real-world study, the median OS was 30 months (130 weeks), compared with 37.3 months in the Checkmate-066 phase III trials on anti-PD-1 antibodies, and 32.7 months in the Keynote-006 study, respectively. However, our patients presented with a 12.5-month (54 weeks) median PFS, which was better than the results of the phase III trials (5.1 and 8.4 months) [5,16,17,18,19]. Our results thus suggest that the survival benefit from PD-1 ICI therapy for AM in routine clinical practice is similar to that was observed in the phase III clinical trials of these agents. [5,16,17,19]. Furthermore, the longer progression-free survival in our cohort suggests the tremendous impact of PD-1 ICI on the prognosis of AM. On the other hand, the ORR was lower (16.8%) in our patient cohort compared to the clinical trial (42%, 41%) [17,18,20].

On the background of the prolonged PFS and low ORR, the small number of patients, who reached CR (6.7%) or PR (10.08%) developed a long-lasting immune response against the tumor, expecting a long-term survival; this also explained why the median prolonged OS was not reached during this study period.

As expected, patients with negative prognostic factors, such as elevated LDH levels, poor performance status (ECOG PS ≥ 2), or brain metastases (stage M1d), may benefit less from PD-1 inhibition. However, in our cohort, the majority of patients had an ECOG PS 0–1 (117, 98.28%), only two patients had an ECOG performance status ≥ 2, and nine (7.56%) patients had an elevated LDH level at the beginning of the anti-PD-1 therapy. Therefore the correlation of the outcome was not significant and explained the prolonged PFS. 

Untreated melanoma brain metastases had a poor prognosis (median survival of 3 to 6 months) [21,22]. Our analysis showed limited benefit among the patients with brain metastases (median OS: 71 weeks). The dual blockade of immune checkpoints with ipilimumab and nivolumab in the clinical trials have shown promising results for patients with asymptomatic brain metastases [23,24]. This will hopefully translate to a survival benefit for this population in everyday clinical care. 

We also investigated whether the administration of anti-PD-1-ICI as a first-, second-, or third line therapy influenced the survival outcome. We observed that those individuals who received PD-1 ICI as a second- or third line treatment benefited more than those who received PD-1 ICI as a first-line therapy. On the background of this difference, it is possible that those patients who had BRAF-positive AM and were previously given BRAF and MEK inhibitors in combination achieved a fast regression of the tumor burden. However, among the BRAF wild-type AM patients who received PD-1 ICI as a second-line treatment, few of them received ipilimumab as first-line therapy and it took significantly more time to achieve a therapeutic response. The majority of BRAF wild-type AM patients who were treated with second-line anti-PD-1 monotherapy had received chemotherapy as the first-line treatment.

According to our results, those patients who harbored BRAF wild-type AM showed a survival benefit when compared to patients with BRAF V600-mutant melanomas. A potential explanation is that patients with BRAF-mutant melanomas were administered BRAF and MEK inhibitor combination therapy or previous BRAF monotherapy (77.4% of the patients with BRAF mutations).

Those few patients who did not receive previous treatment with a BRAF-targeted therapy (5.8%), however, showed less favorable survival outcomes. This finding may be explained by the fact these patients had a high tumor burden and already suffered from symptoms of the disease. Thus, patients receiving a first-line therapy with a BRAF and MEK inhibitor combination achieved a rapid decrease in tumor burden and improvement in their symptoms.

There is an ongoing debate over whether ICI or a BRAF and MEK inhibitor combination should be given as first-line therapy in BRAF V600 mutant AM and if this decision has a significant impact on the long-term survival of the patient. The findings of recent prospective trials (phase II SECOMBIT trial and phase III DREAMSeq trial) resulted in a paradigm shift [4]. Both trials investigated the best possible sequence for patients with therapy-naive stage IV melanoma with a BRAF V600 mutation. Three different therapeutic approaches were compared in the phase II SECOMBIT trial. Patients in the first arm were given encorafenib plus binimetinib until progression, while at the time of progression, they were switched to combined immunotherapy. The second arm was first started on combined immunotherapy until disease progression, which was then switched to combined targeted therapy. In the third arm, encorafenib plus binimetinib were administered for the patients for eight weeks, which was replaced by combined immunotherapy until progression, when they were started on combined targeted therapy. The three-year OS rate was 54%, 62% and 60% in these three arms, while the three-year PFS rate was 41%, 53% and 54%, respectively [4]. The phase III DREAMSeq trial investigated AM patients with a BRAF V600 mutation as well. Here, half of the patients received nivolumab plus ipilimumab (arm A), while the other half was started on dabrafenib plus trametinib (arm B) as first-line treatment. Following disease progression, patients were enrolled into the second phase of the study to receive combined targeted therapy (arm C) or combined immunotherapy (arm D). The two-year OS rate for patients in arm A was 72%, while for arm B it was 52% [4]. These studies revealed that the long-term OS may be prolonged in patients with BRAF V600-mutant tumors who were given a combination of ipilimumab and nivolumab followed by targeted therapy, compared to the reverse scenario [4].

Those patients in our cohort who simultaneously received RT and anti-PD-1 ICI had a higher OS (*p* = 0.010). There is only limited data on the efficacy of RT in patients receiving anti-PD-1 ICI. In a prospective clinical trial, 25 AM patients receiving a combination of RT and anti-PD-1 ICI had better outcomes at the irradiated and also the non-irradiated areas, that could be explained by the abscopal effect [25]. A further study reported that this combination showed significant benefit in 225 patients with advanced mucosal melanoma [26]. Another prospective study assessed a combination of RT and ICI therapy in AM patients who did not respond to anti-PD-1 monotherapy. The abscopal effect could be induced in 38% of the patients [27].

The toxicities from ICI represent a new class of adverse events, referred to as irAEs or immune-mediated adverse events (imAE) that are manageable with early administration of systemic corticosteroids or immunomodulators. In the present study, severe irAEs (grade 3 or 4) in 31.08% of patients were reported, while mild irAEs (grade 1 or 2) occurred in only 15.12% of the patients. These results are in line with the observed frequency of irAE seen in clinical trials [6,15,28]. 

We found that patients with one or more than one irAE (>1 irAE) had significantly longer OS and PFS compared to patients with no irAE. The grade of toxicity also affected the survival, the patients with a grade 3–4 irAE had a significantly improved PFS. These findings suggest the presence of one or more irAEs is an independent predictive marker of PFS and OS. 

Suo et al. showed that, at a 12-week timepoint, the development of irAEs during anti-PD-1 ICI was associated with better treatment outcomes. Weber et al. [28] published a pooled analysis of four clinical trials on nivolumab, where the presence of treatment-related AEs indicated a better ORR. Bastacky et al. [12,28] reported that any irAE was strongly associated with the response to anti-PD-1 ICI, and obesity, but not age or gender, was distinctly associated with irAE development [13]. 

Importantly, the present study has various major limitations, such as its retrospective nature, that result in significant selection bias. In addition, our study is limited by AE reporting being based on the medical documentation; thus, grade 1–2 irAEs could have been underreported.

## 5. Conclusions

Despite the different baseline patient characteristics, the anti-PD-1 monotherapy showed encouraging long-lasting responses in patients with AM in a real-world setting, including long-term outcomes with the potential risk for developing an irAE. This retrospective study demonstrated that the occurrence of one or more irAEs was significantly associated with anti-PD-1 therapy response and they have been shown to be an independent predictive marker of PFS and OS.

## Figures and Tables

**Table 1:** Demographic and clinical characteristics of patient.

	Median (IQR) or N (%)			
	Total Sample * (N = 119)	Non-Responders * (N = 98)	Responders * (N = 20)	*p*-Value **
**Age (years)**	69.0 (57.0–75.0)	68.5 (57.0–75.0)	68.5 (53.5–79.25)	0.622
≥70 years	62 (52.1)	52 (83.9)	10 (16.1)	0.811
<70 years	57 (47.9)	46 (80.7)	10 (17.5)	
**Gender**				
Male	68 (57.1)	54 (79.4)	13 (19.1)	0.466
Female	51 (42.9)	44 (86.3)	7 (13.7)	
**Received doses**	14.0 (7.0–32.0)	12.0 (6.0–26.0)	24.0 (17.5–44.5)	0.002
**Duration of treatment (weeks)**	45.1 (17.3–112.1)	37.6 (16.1–92.3)	118.0 (56.0–192.4)	<0.001
**Primary tumor**				
Occult	21 (17.6)	16 (76.2)	5 (23.8)	0.670
Superficial spreading melanoma (SSM)	23 (19.3)	21 (91.3)	2 (8.7)	
Nodular melanoma (NM)	33 (27.7)	28 (84.8)	5 (15.2)	
Acral lentiginous (ALM)	15 (12.6)	13 (86.7)	2 (13.3)	
Lentigo maligna melanoma (LMM)	2 (1.7)	2 (100.0)	-	
desmoplastic melanoma (DM)	2 (1.7)	2 (100.0)	-	
Not classified	23 (19.3)	16 (69.6)	6 (26.1)	
**T**				0.082
ptx or no primary tumor	35 (29.4)	25 (71.4)	10 (28.6)	
pT1a	3 (2.5)	3 (100.0)	-	
pT2a	6 (5.0)	6 (100.0)	-	
pT2b	5 (4.2)	5 (100.0)	-	
pT3a	9 (7.6)	8 (88.9)	1 (11.1)	
pT3b	16 (13.4)	16 (100.0)	-	
pT4a	9 (7.6)	9 (100.0)	-	
pT4b	36 (30.3)	26 (72.2)	9 (25.0)	
**TIL**				
Brisk	15 (12.6)	12 (80.0)	3 (20.0)	0.316
Non-brisk	12 (10.1)	7 (58.3)	4 (33.3)	
Absent	18 (15.1)	16 (88.9)	2 (11.1)	
Unknown	74 (62.2)	63 (85.1)	11 (14.9)	
**N**				0.158
No	57 (47.9)	51 (89.5)	6 (30.0)	
Yes	58 (48.7)	44 (75.9)	13 (65.0)	
Unknown	4 (3.4)	3 (75.0)	1 (5.0)	
**M** **AJCC 8th edition**				0.136
M0	1 (0.8)	1 (100.0)	-	
M1a	22 (18.5)	19 (86.4)	3 (13.6)	
M1b	15 (12.6)	13 (86.7)	2 (13.3)	
M1c	56 (47.04)	41 (73.2)	14 (25.0)	
M1d	25 (21.0)	24 (96.0)	1 (4.0)	
**Disease stage**				
III	1 (0.8)	1 (100.0)	-	1.000
IV	118 (99.2)	97 (82.2)	20 (16.9)	
**Treatment** NE = 1				
Nivolumab	52 (43.7)	45 (86.5)	6 (11.5)	0.220
Pembrolizumab	66 (55.5)	52 (78.8)	14 (21.2)	
**Line of treatment**				
First	81 (68.1)	66 (81.5)	14 (17.3)	1.000
Second + third	38 (31.9)	32 (84.2)	6 (15.8)	
**Reason for treatment cessation**				
Did not stop	36 (30.3)	24 (66.7)	11 (30.6)	<0.001
AE	13 (10.9)	9 (69.2)	4 (30.8)	
PD	49 (41.2)	48 (98.0)	1 (2.0)	
Exit	19 (16.0)	17 (89.5)	2 (10.5)	
CR	2 (1.7)	-	2 (100.0)	
**IrAE**				
No	64 (53.8)	56 (87.5)	7 (10.9)	0.058
IrAE = 1	24 (20.2)	16 (66.7)	8 (33.3)	
IrAE > 1	31 (26.1)	26 (83.9)	5 (16.1)	
**IrAE toxicity**				
No irAE	66 (55.5)	58 (87.9)	7 (10.6)	
G1-2	37 (31.1)	28 (75.7)	9 (15.4)	0.126
G3-4	16 (13.4)	12 (75.0)	4 (25.0)	
**Brain metastases**				
No	94 (79.0)	74 (78.7)	19 (20.2)	0.070
Yes	25 (21.0)	24 (96.0)	1 (4.0)	
**Lactate dehydrogenase (LDH)**				
Normal LDH	110 (92.4)	90 (81.8)	19 (17.3)	0.706
Elevated LDH	9 (7.6)	8 (88.9)	1 (11.1)	
**BRAF**				
Wild type	88 (73.9)	70 (79.5)	17 (19.3)	0.272
Positive	31 (26.1)	28 (90.3)	3 (9.7)	
	**Total sample (N = 31)**	**Non-responders (N = 28)**	**Responders (N = 3)**	
**BRAF mutation genotype**				
V600E	22 (71.0)	20 (90.9)	2 (9.1)	1.000
V600K	8 (25.8)	7 (87.5)	1 (12.5)	
V600 others	1 (3.2)	1 (100.0)	-	

CR—complete response, PR—partial response, SD—stable disease, PD—progressive disease, LDH—lactate dehydrogenase, AJCC—American Joint Committee on Cancer. * The non-responder (stable disease and progressive disease) and responder (partial and complete responses) subgroups were created based on the therapy responses and only one responder was excluded since the therapy response was NE (not evaluable). ** Mann–Whitney U test or Fisher’s exact test. A *p*-value < 0.05 was considered statistically significant.

**Table 2:** Brain metastases.

	Total Sample (N = 25)	Non-Responders (SD + PD) (N = 24)	Responders (CR + PR) (N = 1)	
**Brain metastases—Treatment**				
Stereotaxy	13 (52.0)	12 (50.0)	1 (100.0)	0.916
WBRT	7 (28.0)	7 (29.2)	-	
Both	3 (12.0)	3 (12.5)	-	
No treatment	1 (4.0)	1 (4.2)	-	
Operation	1 (4.0)	1 (4.2)	-	

CR—complete response, PR—partial response, SD—stable disease, PD—progressive disease, WBRT—whole brain radiation therapy. The non-responder (stable disease and progressive disease) and responder (partial and complete responses) subgroups were created based on the therapy responses and only one responder was excluded since the therapy response was NE (not evaluable). Mann–Whitney U test or Fisher’s exact test. A *p*-value < 0.05 was considered statistically significant.

**Table 3:** Response rates to nivolumab or pembrolizumab.

	All Patients				BRAF Positive		BRAF Wild Type		
Responses	Total (N = 119)	First-Line Treatment (N = 81)	≥Second- Line Treatment (N = 38)	Total N = 31	First-Line Treatment (N = 7)	≥Second-Line Treatment (N = 24)	Total (N = 88)	First-Line Treatment (N = 74)	≥Second-Line Treatment (N = 14)
CR	8 (6.72%)	3 (3.7%)	5 (13.15%)	2 (6.45%)	0	2 (8.33%)	6 (6.81%)	3 (4.05%)	3 (21.42%)
PR	12 (10.08%)	11 (13.5%)	1 (2.63%)	1 (3.22%)	0	1 (4.16%)	11 (12.5%)	11 (14.86%)	0
SD	42 (35.28%)	28 (34.56%)	14 (36.84%)	10 (32.2%)	1 (14.28%)	9 (37.5%)	32 (36.36%)	27 (36.48%)	5 (35.71%)
PD	56 (47.04%)	38 (46.9%)	18 (47.36%)	18 (58.06%)	6 (85.71%)	12 (50%)	38 (43.18%)	32 (43.24%)	6 (42.85%)
NE	1 (0.84%)	1 (1.23%)	0	0	0	0	1 (1.13%)	1 (1.35%)	0
DCR *	52.08%	51.76%	52.62%	41.87%	14.28%	49.99%	55.67%	55.39%	35.71%
ORR **	**16.8%**	**17.2%**	**15.78%**	**9.67%**	**14.28%**	**12.49%**	**19.31%**	**18.91%**	**21.42%**

* CR = complete responses; DCR = disease control rate; NA = not available; PD = progressive diseases; ORR = objective response rate; PR = partial responses; SD = stable diseases * DCR = CR + PR + SD ** ORR = CR + PR.

**Table 4:** Survival analysis of the investigated patients.

		N	Median OS (Weeks)	LCI	UCI	*p*-Values (Log-Rank)	Median PFS (Weeks)	LCI	UCI	*p*-Values (Log-Rank)
**All patients (median)**		**119**	130.29	81.02	179.57	-	54.86	19.75	89.97	-
Age (years)	<70	62	135.14	81.05	189.23	0.324	54.57	1.75	107.40	0.982
	≥70	57	86.00	8.31	163.69		54.86	17.97	91.75	
Gender	Male	68	139.43	58.53	220.33	0.304	97.14	14.38	179.90	0.139
	Female	51	77.29	36.17	118.42		46.86	27.59	66.13	
T	ptx or occult primary tumor	35	229.430	86.48	372.38	**0.020**	143.86	0.00	293.85	**0.003**
	pT1a	3	86.00	22.68	149.33		43.14	36.29	49.99	
	pT2a	6	24.00	16.80	31.20		13.86	7.34	20.38	
	pT2b	5	142.86	-	-		30.57	0.00	69.82	
	pT3a	9	-	-	-		54.00	48.57	59.44	
	pT3b	16	48.43	0.00	145.11		35.71	19.76	51.66	
	pT4a	9	-	-	-		80.43	0.00	190.64	
TIL	Unknown	74	86.00	42.27	129.73	0.381	42.71	26.02	59.40	0.285
	Brisk	15	229.43	9.08	449.78		229.43	0.00	502.56	
	Non-brisk	12	142.86	15.73	269.99		80.43	-	-	
	Absent	18	-	-	-		97.14	-	-	
N	No	57	139.43	36.68	242.18	0.289	87.57	33.31	141.83	0.179
	Yes	58	103.14	22.90	183.38		43.00	22.75	63.26	
	Unknown	4	32.14	27.24	37.04		17.29	5.11	29.47	
M AJCC 8th edition	M0	1	61.71	-	-	0.109	27.14	-	-	0.085
	M1a	22	57.00	16.54	97.47		37.86	21.45	54.27	
	M1b	15	-	-	-		-	-	-	
	M1c	56	178.57	86.87	270.27		107.43	0.00	228.49	
	M1d	25	52.57	0.00	134.24		35.29	13.53	57.05	
Line of treatment	First	81	94.14	33.01	155.27	0.596	46.86	30.03	63.69	0.175
	Second + third	38	178.57	20.51	336.63		172.00	0.00	382.17	
Treatment NA = 1	Nivolumab	52	86.00	19.33	152.68	0.358	43.14	23.74	62.54	0.606
	Pembrolizumab	66	139.71	67.66	211.76		61.43	4.67	118.19	
irAE	No	64	46.43	34.53	58.33	**<0.001**	27.86	19.92	35.80	**<0.001**
	IrAE = 1	24	142.86	52.49	233.23		107.43	0.00	245.26	
	>1 IrAE	31	258.00	-	-		-	-	-	
BRAF	Wild-type	88	130.29	80.80	179.78	0.161	58.14	19.19	97.09	0.462
	Positive	31	71.71	0.00	172.50		35.29	0.00	82.81	
Brain metastases	No	94	135.14	57.31	212.97	0.259	61.00	4.44	117.56	0.063
	Yes	25	71.71	0.00	169.70		36.00	7.57	64.43	
Response of the treatments	PD + SD	98	71.71	40.01	103.42	**<0.001**	42.14	31.64	52.64	**<0.001**
	CR + PR	20	-	-	-		-	-	-	
Received radiation therapy	No	86	86.00	21.93	150.07	**0.010**	43.14	22.36	63.92	0.326
	Yes	33	-	-	-		80.43	25.14	135.72	
**BRAF-wild type (*n* = 88)**										
Line of treatment	First	74	103.14	41.07	165.21	0.392	57.00	19.62	94.38	0.218
	Second + third	14	229.43	43.62	415.25		229.43	-	-	
**BRAF V600 mutation (*n* = 31)**										
Line of treatment	First	7	69.14	0.00	159.09	0.312	12.29	6.77	17.81	**0.022**
	Second + third	24	71.71	0.00	222.94		61.43	0.00	225.52	

CR—complete response, PR—partial response, SD—stable disease, PD—progressive disease, LDH—lactate dehydrogenase, NA—not available, AJCC—American Joint Committee on Cancer; LCI = lower bound of the 95% confidence interval; PD + SD = progressive disease and stable disease; PR + CR = partial responders and complete responders; UCI = upper bound of the 95% confidence interval. A *p*-value < 0.05 was considered statistically significant.

**Table 5:** Cox regression analysis of the patients.

	Survival		Progression-Free Survival	
	HR for Death (OS) (95% CI)	*p*-Value	HR for Progression-Free (PFS) (95% CI)	*p*-Value
Age group/70 years+	1.66 (0.96–2.88)	0.072	1.12 (0.67–1.86)	0.675
Gender/female	1.16 (0.69–1.96)	0.570	1.23 (0.74–2.02)	0.423
Line of treatment/advanced setting	1.04 (0.53–2.06)	0.906	0.50 (0.24–1.03)	0.060
IrAE		0.000		0.000
IrAE = 1	0.31 (0.14–0.65)	**0.002**	0.31 (0.15–0.62)	**0.001**
IrAE > 1	0.21 (0.10–0.44)	**0.000**	0.17 (0.08–0.35)	**0.000**
M		0.119		0.501
M/M1a	0.38 (0.04–3.27)	0.375	0.34 (0.04–2.91)	0.325
M/M1b	0.10 (0.01–1.08)	0.058	0.16 (0.02–1.63)	0.123
M/M1c	0.26 (0.03–2.26)	0.221	0.27 (0.03–2.33)	0.232
M/M1d	0.41 (0.02–8.94)	0.567	0.21 (0.01–4.35)	0.312
BRAF/positive	1.96 (1.02–3.77)	**0.044**	2.68 (1.26–5.72)	**0.011**
Brain metastases/yes	0.84 (0.09–7.73)	0.880	1.62 (0.18–14.25)	0.666
Therapy responses/CR + PR	0.14 (0.05–0.43)	**0.001**	0.09 (0.03–0.30)	**0.000**
Grade of irAE		0.154		0.105
Grade of irAE/G1-G2	0.26 (0.05–1.40)	0.116	0.32 (0.06–1.72)	0.184
Grade of irAE/G3-G4	0.14 (0.02–1.03)	0.053	0.11 (0.01–0.89)	**0.038**
Received radiation therapy/yes	0.48 (0.24–0.98)	**0.043**	0.85 (0.47–1.51)	0.573

CI = confidence interval; HR = hazard ratio; CR + PR = complete response and partial response; *p*-value < 0.05 was considered statistically significant.

## Data Availability

The data presented in this study are available within the article. Further data are available on request from the corresponding author.
